# CDK4/6 inhibitor–statin interaction and rhabdomyolysis in breast cancer treatment: a case-based systematic review

**DOI:** 10.1007/s00280-026-04913-w

**Published:** 2026-07-01

**Authors:** Rafael Batista João, Leonardo Ribeiro Soares, Paulo César Ragazzo, Ruffo Freitas-Junior

**Affiliations:** 1 Neurology and Neurophysiology Department, Goiânia Neurological Institute, Goiânia, Goiás Brazil; 2 CORA – Advanced Center for Diagnosis of Breast Diseases, School of Medicine and Hospital das Clínicas, Federal University of Goiás, Goiás, Brazil; 3 IMO Mastology and Oncology Institute, Goiânia, Goiás Brazil; 4 Gynecology and Breast Department, Araújo Jorge Cancer Hospital, Goiás Anticancer Association, Goiânia, Goiás Brazil

**Keywords:** Breast cancer, Rhabdomyolysis, CDK4/6 inhibitors, Statins, Drug–drug interaction, CYP3A4, OATP1B1, Myopathy

## Abstract

**Purpose:**

Cyclin-dependent kinase (CDK) 4/6 inhibitors have significantly advanced the treatment of hormone receptor-positive, HER2-negative metastatic breast cancer, improving outcomes. However, their concurrent use with statins, a widely prescribed class of drugs, may pose a risk of rhabdomyolysis due to pharmacokinetic and pharmacodynamic interactions. This systematic review analyzes reported cases to characterize clinical presentations, therapeutic approaches, and outcomes.

**Methods:**

A systematic search of PubMed, Embase, Scopus, and Web of Science from inception to January 2026 for case reports and case series documenting rhabdomyolysis in patients treated with CDK4/6 inhibitors. Data on demographics, clinical features, laboratory findings, management strategies, and outcomes were extracted. Quality assessment of the included cases was performed using the Joanna Briggs Institute Critical Appraisal Checklist.

**Results:**

Seven case reports involving female patients aged 55–81 years were analyzed. All patients were treated for metastatic breast cancer with CDK4/6 inhibitors (ribociclib, palbociclib, or abemaciclib) alongside statins (simvastatin, atorvastatin, or rosuvastatin). Rhabdomyolysis onset ranged from 3 days to 48 months after initiation of combination therapy, or shortly after statin dose escalation in the setting of long-term CDK4/6 inhibitor use. Clinical presentations included severe myalgia, muscle weakness, and dark urine, with creatine kinase levels ranging from 3,070 to 47,000 U/L. Acute kidney injury was reported in five cases. Management primarily involved cessation of the implicated drugs and aggressive hydration, with adjunctive treatments such as corticosteroids, plasma exchange, or intravenous immunoglobulin. Five patients recovered fully, one had partial recovery with persistent renal dysfunction, and one fatality was reported.

**Conclusions:**

Rhabdomyolysis due to CDK4/6 inhibitor–statin interactions is a rare but potentially life-threatening complication. Vigilant monitoring, timely intervention, and tailored treatment strategies are essential for preventing complications and improving patient outcomes.

**Supplementary Information:**

The online version contains supplementary material available at 10.1007/s00280-026-04913-w.

## Introduction

Pharmacotherapy safety requires vigilant detection and reporting of new adverse drug events and drug–drug interactions [1]. Palbociclib, ribociclib, and abemaciclib are cyclin-dependent kinase 4/6 (CDK4/6) inhibitors that have changed the treatment paradigm for hormone receptor-positive, HER2-negative metastatic breast cancer [2]. Selective inhibition of CDK4 and CDK6 in tumor cells blocks the G1/S cell-cycle transition, inhibiting tumor growth and improving survival [3, 4]. Although CDK4/6 inhibitors are generally well tolerated, inhibition of the CYP3A4 enzyme by CDK4/6 inhibitors increases the serum levels of other drugs metabolized by this pathway [5]. Statins, widely used to manage hyperlipidemia and to reduce cardiovascular risk, include commonly prescribed agents metabolized via CYP3A4 [6].

CDK4/6 inhibitors also modulate the action of organic anion-transporting polypeptides (OATPs), such as OATP1B1 and OATP1B3 [7], fundamental to hepatic statin uptake and clearance [7–9]. Therefore, the coadministration with CDK4/6 inhibitors can cause statin accumulation, which increases the risk of myopathy, including rhabdomyolysis and its complications [5, 10]. This systematic review compiles case reports of rhabdomyolysis associated with CDK4/6 inhibitor–statin interactions, emphasizing clinical manifestations, therapeutic approaches, and outcomes. By integrating case-level clinical patterns with mechanistic insights, we aim to support clinicians in recognizing, preventing, and managing this rare but potentially severe adverse event.

## Methods

We performed and reported this systematic review following the Cochrane Handbook for Systematic Reviews of Interventions [11] and the Preferred Reporting Items for Systematic Reviews and Meta-Analyses (PRISMA) statement [12]. The protocol was registered prospectively at PROSPERO (Registration ID: CRD42025631033).

### Inclusion criteria

We considered for inclusion case reports or case series meeting the following criteria:

1. Patient Population: Breast cancer patients treated with CDK4/6 inhibitors (ribociclib, palbociclib, or abemaciclib) concomitantly with statins;
2. Diagnosis of rhabdomyolysis: Cases documenting rhabdomyolysis defined by one or more of the following:

- Clinical Criteria: Symptoms such as severe muscle pain, weakness, or dark urine;
- Laboratory Criteria: Elevated creatine kinase (CK) levels > 1,000 U/L;
- Complications: Evidence of acute kidney injury (AKI) attributed to rhabdomyolysis (e.g., myoglobinuria, elevated serum creatinine).

3. Data Reporting Requirements: Cases providing sufficient clinical-demographics and therapeutic information, including:

- Clinical-demographics (age, sex, comorbidities);
- Detailed drug regimens (type, dosage, and duration of CDK4/6 inhibitors and statins, as well as co-medications);
- Clinical presentation and laboratory findings relevant to rhabdomyolysis;
- Therapeutic interventions (e.g., cessation of medications, hydration, use of corticosteroids or plasma exchange);
- Outcomes (e.g., recovery, mortality).

4. Language Restrictions: No language restrictions were applied; articles in languages other than English would have been translated if necessary;
5. Temporal Scope: No restrictions on publication date were applied to capture all available data.

### Exclusion criteria

We excluded studies meeting the following criteria:

1. Cases lacking sufficient detail to attribute rhabdomyolysis to CDK4/6 inhibitor–statin interaction;
2. Reports with incomplete or unclear data on clinical presentation, therapeutic interventions, or outcomes;
3. Duplicate case reports;
4. Studies not providing primary case data (e.g., reviews, commentaries, or editorials).

A summary table with inclusion and exclusion criteria is provided in the supplementary material (Supplemental Table 1).

### Search strategy and data extraction

We systematically searched PubMed, Embase, Scopus, and Web of Science from inception to January 2026. The exact search strategy used is detailed in the supplementary material (Supplemental Table 2). Two authors (R.J. and L.S.) independently screened studies according to the predefined search criteria and extracted data to a standardized spreadsheet. Disagreements between these authors were resolved by consensus with a third author. In addition, we manually reviewed the references lists of all included and previous relevant studies (backward citation tracking) and screened the articles citing the included studies (forward citation tracking) [13]. The extracted data included: (1) patient demographics (e.g., age, sex, comorbidities); (2) drug regimens (e.g., type and dosage of CDK4/6 inhibitors and statins, co-medications such as fulvestrant or aromatase inhibitors); (3) clinical manifestations (e.g., timing of symptoms, creatine kinase levels, renal impairment); (4) therapeutic interventions (e.g., drug cessation, hydration, corticosteroids, plasma exchange); and (5) outcomes (e.g., recovery, mortality). All records considered potentially eligible after title and abstract screening were retrieved for full-text review.

### Endpoints

Our endpoints were the detailed clinical characterization of rhabdomyolysis (including CK levels, complications, and timing of onset), therapeutic interventions, and patient outcomes.

### Quality assessment

We assessed the quality of the included case reports using the Joanna Briggs Institute Critical Appraisal Checklist for Case Reports (JBI) [14]. This tool evaluates reports across eight domains: (1) the clear description of the patient’s demographic characteristics; (2) a clearly described and appropriately presented patient history, ideally as a timeline; (3) a clear description of the patient’s clinical condition at the time of presentation; (4) clear reporting of diagnostic tests or assessment methods and their results; (5) a detailed description of the intervention(s) or treatment procedure(s); (6) the clear reporting of the post-intervention clinical condition; (7) identification and description of adverse events (harms) or unanticipated events; and (8) whether the case report provided takeaway lessons relevant to clinical practice. Each case report was scored as “Yes” (Y), “No” (N), or “Unclear” (U) for each domain.

## Results

### Studies selection and characteristics

**Fig. 1:**
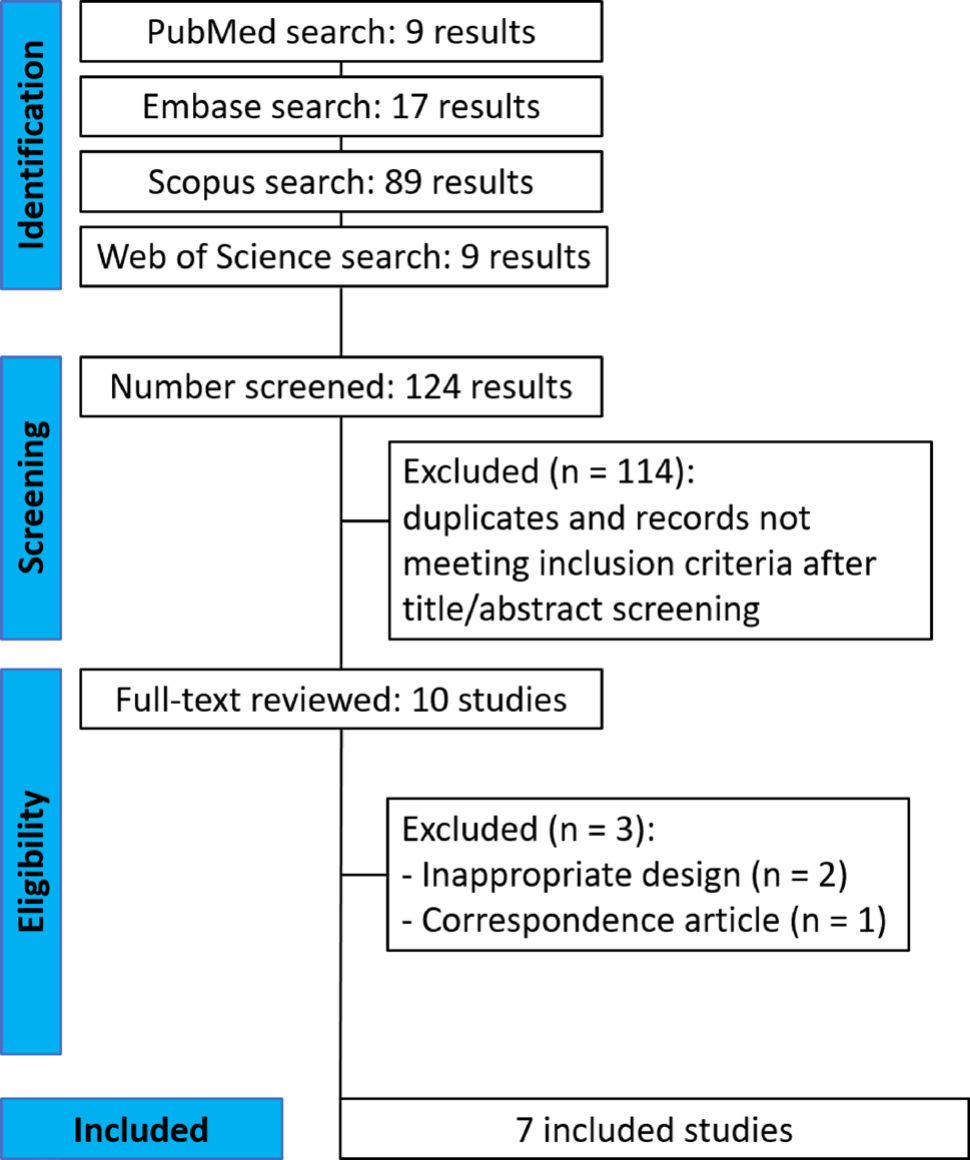
PRISMA flow diagram of study screening and selection

As shown in Fig. 1, we identified 124 results through the search strategy. Ten studies were assessed in full after removing duplicates and excluding studies based on title and abstract review. Of these, seven case reports were included for analysis (Fig. 1) [15–21]. No additional studies were identified for inclusion based on the forward/backward citation tracking method.

### Years and countries of published cases

The studies included in this review were published between 2017 and 2025 and reported from Israel [15], Australia [16], France [17, 18], Denmark [19], and the United States of America [20, 21].

### Cases characteristics

All the cases were of female patients with ER positive, HER2 negative metastatic breast cancer following the conventional therapy protocols and who developed rhabdomyolysis during the use of CDK4/6 inhibitors along with statins. The age ranged from 55 to 81 years [15–21]. Ribociclib (dose range: 200–600 mg/day) was used in three cases [15, 16, 18], palbociclib (125 mg/day) in three cases [17, 19, 20], and abemaciclib (150 mg twice daily) in one case [21]. Other drugs include aromatase inhibitors such as letrozole (2.5 mg/day) in four cases [15, 17, 18, 21], anastrozole (1 mg/day) in one case [16], and fulvestrant (500 mg/day) in two cases [19, 20].

### Time to onset and clinical presentation

The time to onset of rhabdomyolysis symptoms varied substantially, from three days [19] to 48 months [16] after initiation of concomitant therapy; in one case, onset followed statin dose escalation during long-term abemaciclib use [21]. Common symptoms included muscle pain, profound weakness, and dark-colored urine, classic hallmarks of rhabdomyolysis. Laboratory investigations revealed CK levels ranging from 3,070 U/L [15] to 47,000 U/L [17]. AKI was diagnosed in five of the seven cases due to myoglobinuria-induced nephrotoxicity [15, 16, 18, 19, 21].

### Therapeutic interventions

Management strategies were primarily directed at discontinuation of the implicated CDK4/6 inhibitor and statin, combined with hydration [17–21]. Additional interventions varied according to clinical severity. Corticosteroids were employed in two cases [17, 20], while intravenous immunoglobulin (IVIG) and plasma exchange (PlEx) were used in two distinct cases [17, 19].

### Outcomes

Five of the seven patients achieved full recovery following timely diagnosis and intervention [15–19]; one patient experienced partial recovery with persistent renal dysfunction [21], and one fatal outcome was reported [20]. Among the recovered patients, ribociclib was cautiously reintroduced in two cases under vigilant monitoring, while statins were permanently discontinued [15, 18]. Conversely, palbociclib was definitively withdrawn in one case, with the therapeutic regimen modified to tamoxifen and capecitabine after disease progression [17]. For two cases, the therapeutic strategies upon discharge were not specified [16, 19]. Despite aggressive hydration, corticosteroid therapy, and intensive care, one patient progressed to a fatal outcome after developing severe rhabdomyolysis and AKI [20].

### Statin implications

Four patients were treated with simvastatin (dose range: 20–40 mg/day) [15, 17–19], two with rosuvastatin (10–40 mg/day) [16, 21], and one with atorvastatin (40 mg/day), which was involved in the fatal outcome [20].

### Additional contributing factors

In the case reported by Poumeaud et al., the patient was also on fenugreek for two days before the onset of the acute myopathy. Fenugreek is an herbal supplement reported to inhibit CYP3A4 activity [17].

### Additional myopathy and pharmacogenetic investigations

Three cases included extensive autoimmune panel testing (including Anti-HMG-CoA-reductase-IgG) as part of the diagnostic workup for rhabdomyolysis, which were negative for other causes of acute myopathy [16, 18, 19]. Additionally, in one case co-medicated with simvastatin, genetic analysis revealed the heterozygous presence of the common SNP rs4149056 in the SLCO1B1 gene, associated with altered statin metabolism and an increased risk of statin-induced myopathy [19]. The main characteristics of the included cases are summarized in Table 1.

**Table 1:** Main features of the included cases (Positive estrogen-receptors, HER-2 negative, metastatic breast cancer)

Authors (year) Country	Gender, Age	CDK 4/6 inhibitor	Co-medication	Statin	Time to onset of symptoms	CK peak (U/L)	AKI	Therapeutic strategies during Rhabdomyolysis	Myopathy Outcome	Therapeutic Strategies Upon Discharge
Kim et al. (2025) *USA*	Female, 66y	Abemaciclib, dose N/A	Fulvestrant, dose N/A	Rosuvastatin, 20 → 40 mg/day	Weeks after statin dose escalation†	> 39,000	Yes	Abemaciclib and Rosuvastatin cessation; Hydration; Hemodialysis	Partial recovery; Persistent dialysis-dependence	Abemaciclib reintroduced; Statin definitively discontinued
Badran et al. (2023) *Israel*	Female, 73y	Ribociclib, 600 mg/day, Cycle N/A	Letrozole, Dose N/A	Simvastatin, 40 mg/day	16 months†	3,070	Yes	Ribociclib and Simvastatin cessation; Hydration	Full recovery	Ribociclib re-introduced; Simvastatin definitively discontinued
Teo et al. (2023) *Australia*	Female, 55y^*^	Ribociclib, 200 mg/day, 21/28 days	Anastrozole, 1 mg/day	Rosuvastatin, 10 mg/day	48 months†	3,608	Yes	Rosuvastatin cessation; Hydration	Full recovery	*Non-reported*
Poumeaud et al. (2022) *France*	Female, 81y	Palbociclib, 125 mg/day, 21/28 days	Letrozole, 2,5 mg/day	Simvastatin, 20 mg/day	23 days	47,000	N/A	Palbociclib and Simvastatin cessation; Hydration; Corticosteroids, PlEx	Full recovery	Palbociclib definitively discontinued; Switch of letrozole to tamoxifen and capecitabine introduction after disease progression
Streicher et al. (2020) *France*	Female, 68y	Ribociclib, 600 mg/day, 21/28 days	Letrozole 2,5 mg/day	Simvastatin, 40 mg/day	21 days	37,000	Yes	Ribociclib and Simvastatin cessation; Hydration	Full recovery	Ribociclib re-introduced; Simvastatin definitively discontinued
Nersesjan et al. (2019) *Denmark*	Female, 71y	Palbociclib, 125 mg/day, 21/28 days	Fulvestrant, 500 mg/day, Day 1 and 14	Simvastatin, 40 mg/day	3 days	> 22,000	No	Palbociclib, Fulvestrant and Simvastatin cessation; Hydration; IVIG	Full recovery	*Non-reported*
Nelson et al. (2017) *USA*	Female, 63y^*^	Palbociclib, 125 mg/day, Cycle N/A	Fulvestrant, Dose N/A	Atorvastatin, 40 mg/day	56 days†	14,572	Yes	Palbociclib, Fulvestrant and Atorvastatin cessation; Hydration; Corticosteroids	Fatal	-

y: years of age; ^*^ - Approximated age cited in the report; HER2: Human Epidermal growth factor Receptor-type 2; CDK 4/6: Cyclin-dependent kinase 4/6; N/A: Not available;† - Approximated time reported; CK: Creatine Kinase; U/L: Units per liter; AKI: Acute Kidney Injury; PlEx: Plasma exchange; IVIG: Intravenous immunoglobulin

### Quality assessment

The quality appraisal based on the JBI critical appraisal checklist [14] indicated overall low concern across the assessed reporting domains. While all cases satisfied the majority of checklist items, some reports presented unclear responses in specific areas. Two reports had unclear responses for Q5 (description of intervention or treatment procedures), one report for Q6 (post-intervention clinical condition), and one report for Q8 (takeaway lessons relevant to clinical practice). The summarized data are shown in Table 2.

**Table 2:** Reported cases and their reporting quality according to the Joanna Briggs Institute Critical Appraisal Checklist for Case Reports

Study, year	Q1	Q2	Q3	Q4	Q5	Q6	Q7	Q8
Kim et al. 2025	Y	Y	Y	Y	Y	Y	Y	Y
Badran et al. 2023	Y	Y	Y	Y	Y	Y	Y	U
Teo et al. 2023	Y	Y	Y	Y	U	U	Y	Y
Poumeaud et al. 2022	Y	Y	Y	Y	Y	Y	Y	Y
Streicher et al. 2020	Y	Y	Y	Y	Y	Y	Y	Y
Nersesjan et al. 2019	Y	Y	Y	Y	Y	Y	Y	Y
Nelson et al. 2017	Y	Y	Y	Y	U	Y	Y	Y

Questions: Q1. Were the patient’s demographic characteristics clearly described? Q2. Was the patient’s history clearly described and presented as a timeline? Q3. Was the current clinical condition of the patient on presentation clearly described? Q4. Were diagnostic tests or assessment methods and the results clearly described? Q5. Was the intervention(s) or treatment procedure(s) clearly described? Q6. Was the post-intervention clinical condition clearly described? Q7. Were adverse events (harms) or unanticipated events identified and described? Q8. Does the case report provide takeaway lessons?Y = Yes; N = No; U = Unclear; N/A = Not applicable

## Discussion

The CDK4/6 inhibitors’ interaction with statins demonstrates the difficulties of dealing with polypharmacy in breast cancer therapy [22, 23]. Such drug combinations are particularly concerning because patients with metastatic breast cancer often have comorbidities requiring lipid-lowering therapies [24]. Moreover, the use of aromatase inhibitors as a co-medication may increase the need for dyslipidemia treatment [25, 26]. This drug-drug interaction is partially underpinned by CYP3A4 enzyme inhibition by CDK4/6 inhibitors. Of note, simvastatin and atorvastatin are known to be metabolized via CYP3A4 to a large extent and, therefore, are at high risk of having their pharmacokinetics altered by CDK4/6 inhibitors [6], which can lead to statin accumulation and consequently enhanced risk of adverse events, such as rhabdomyolysis [27, 28].

From a pathogenic perspective, rhabdomyolysis in this context results from multiple related mechanisms, including drug-induced toxicity, drug pharmacokinetics, and drug pharmacodynamics [29]. Statins inhibit HMG-CoA, thereby decreasing the intracellular cholesterol synthesis. However, this inhibition also affects other vital pathways for the muscle cell membrane integrity and energy metabolism such as those involving isoprenoids and coenzyme Q10 [30, 31]. In addition, statins have been shown to enhance the activity of the muscle-specific ubiquitin-proteasome system (a non-lysosomal intracellular protein degradation system) which may affect the myocyte cell membrane [32]. Therefore, statins can affect mitochondrial function, enhance oxidative stress and induce myocyte apoptosis [33].

While lipophilic statins like simvastatin and atorvastatin are highly effective in reducing cardiovascular risk, their dependence on CYP3A4 metabolism may limit their co-administration with CDK4/6 inhibitors [15–20]. Importantly, this synergistic toxicity may not be limited to CYP3A4 metabolism-dependent statins. Two of the included cases, involving rosuvastatin, which is primarily metabolized through alternative pathways, such as CYP2C9, suggest that additional pharmacodynamic interactions or individual susceptibilities may contribute to statins-induced myopathy risk [16, 21, 34, 35]. Thus, even statins metabolized by alternate pathways may interact through non-CYP3A4-mediated mechanisms, emphasizing the need for close patient monitoring during polypharmacy [36].

Although all three CDK4/6 inhibitors are linked to CYP3A-dependent disposition, their interaction profiles are not identical [37]. Palbociclib is a weak time-dependent inhibitor of CYP3A [38], whereas ribociclib shows a stronger inhibitory effect on CYP3A, with dose-dependent interaction potential [39]. Abemaciclib depends on CYP3A metabolism, with active metabolites additionally contributing to its overall pharmacokinetic profile [40]. These differences support a more individualized assessment of drug–drug interaction risk [37].

Furthermore, CDK4/6 inhibitors also affect the function of OATPs (e.g., OATP1B1 and OATP1B3) which are involved in hepatic uptake and clearance of statins [8]. For instance, rosuvastatin is a substrate for OATP1B1, a transporter involved in hepatic uptake from the portal circulation [35]. Ribociclib, by inhibiting OATP1B1 function, may increase the bioavailability of rosuvastatin, thereby increasing the risk of adverse events [8, 36]. In addition, polymorphisms in the SLCO1B1 gene, which encodes OATP1B1, may influence the metabolism of statins. The single nucleotide polymorphism (SNP) rs4149056 is associated with reduced transporter function and consequent enhancement of the risk of myopathy with statin use [35]. Interestingly, Nersesjan et al. identified this polymorphism in their patient receiving simvastatin alongside palbociclib; this is a real-life scenario showing that genetic factors can enhance the risk of adverse events during CDK4/6 inhibitor-statin drug interactions [19].

Considering these interactions and pharmacogenetic factors, the lipid-lowering therapeutic strategy in this setting could include non-statin drugs, such as ezetimibe and PCSK9 inhibitors. These agents circumvent OATP-mediated mechanisms and CYP450 metabolism and therefore may be especially suitable for patients with high cardiovascular risk [43, 44]. Potential herb–drug interactions should also be considered. The fenugreek use described in the case report by Poumeaud et al., was identified by the authors as a possible contributing factor due to its quercetin content, which is a strong CYP3A4 inhibitor. Although the short duration of concomitant use may have limited its impact, fenugreek likely reduced the metabolism of both palbociclib and simvastatin, compounding the risk of adverse events[17]. This additional contributing factor exemplifies the importance of identifying all substances patients consume, including supplements and over-the-counter medications [45–48]. It also highlights the complexity of managing polypharmacy in oncological care, where even seemingly innocuous substances may significantly alter drug pharmacokinetics [45–47, 49].

Beyond pharmacokinetics, CDK4/6 inhibitors can also affect myocyte vulnerability through modulation of cell cycle and repair processes in skeletal muscle cells [50]. CDK4/6 inhibition impairs cell-cycle progression at the G1/S transition point [5]; this may affect the recovery of muscle fibers following subclinical injury by stunting the regenerative processes [50]. Individualized dose adjustments of CDK4/6 inhibitors should be considered for individuals with predisposing factors for toxicity, such as advanced age, comorbidities, or concurrent use of high-risk medications [8, 51]. These recommendations align with the therapeutic strategies identified in the reviewed cases, which involved chemotherapy adjustments and cessation or substitution of implicated drugs [15–21].

CDK4/6 inhibitors-statin interactions involve a complex interplay of pharmacokinetics, pharmacodynamics, and cellular vulnerabilities. In summary, CYP3A4 inhibition by CDK4/6 inhibitors leads to statin accumulation due to reduced metabolism of these drugs [6], while altered OATP1B1 and OATP1B3 activity reduces statins hepatic clearance, further increasing systemic exposure [8]. Pharmacodynamically, statins disrupt muscle cell membrane stability and energy metabolism by inhibiting HMG-CoA reductase, impairing mitochondrial function, and upregulating the ubiquitin-proteasome pathway [30–33]. Moreover, CDK4/6 inhibitors may exacerbate the risk of myopathy by delaying regenerative responses to myocyte injury through G1/S phase arrest [4, 52]. In this context, genetic polymorphisms, such as SLCO1B1 variants, may further heighten susceptibility to genetic-drug–drug interaction and subsequent statin accumulation [35]. Across the included reports, a causal relationship between CDK4/6 inhibitor–statin coadministration and rhabdomyolysis is supported by converging clinical–pharmacological features. From a pharmacovigilance perspective, these elements are consistent with established causality frameworks such as the World Health Organization–Uppsala Monitoring Centre (WHO-UMC) system and the Naranjo algorithm [41, 42]. Not all included reports provided a formal causality assessment, and case-level detail was not uniform across reports. Nevertheless, the available evidence supports key domains of causality assessment, including temporal association, clinical improvement after drug withdrawal (dechallenge), and exclusion of alternative causes. Rechallenge data are limited, as expected for severe adverse events such as rhabdomyolysis, although partial reintroduction of CDK4/6 inhibitors has been reported in selected cases under close monitoring.

The pathological hallmark of rhabdomyolysis (e.g., extensive myocyte necrosis) results in the release of intracellular components, including CK, myoglobin, and electrolytes such as potassium [28]. Myoglobin’s nephrotoxicity, leading to tubular obstruction, oxidative injury, and AKI was evident in most of the reviewed cases [15, 16, 18, 20, 21]. In addition, three of the seven reported cases complemented the investigations with autoimmune myopathy-related antibodies panels (including Anti-HMG-CoA-reductase-IgG antibodies), which were negative [16, 19, 20]. Six patients recovered with intervention [15–19, 21] but one fatal outcome was reported [20]. The fatal case, reported by Nelson et al., demonstrated how the quick onset and progression of clinical manifestations, such as muscle pain, weakness, and dark urine, can escalate into irreversible complications despite intensive care [20].

Proactive monitoring is essential in terms of prevention. Before combining CDK4/6 inhibitors and statins, baseline CK levels, kidney function tests, and liver function tests should be obtained [15, 53, 54, 55]. Pharmacogenomic testing may identify genetic factors associated with increased risks of non-favourable events [35]. The onset of myopathy symptoms varied between days and years among the cases, highlighting the importance of early recognition and monitoring throughout treatment follow-up. Patients should be informed about the signs and symptoms of acute myopathy, such as pain and weakness; this may facilitate earlier reporting and prompt management before severe complications develop. Importantly, other agents frequently used in breast cancer treatment settings, such as taxanes, anthracyclines, corticosteroids, and potent CYP3A4 inhibitors (e.g., ritonavir, ketoconazole), may alter muscle metabolism or drug bioavailability and occasionally lead to skeletal muscle toxicity. However, the underlying mechanisms differ from the pharmacokinetic and transporter-mediated interaction observed with CDK4/6 inhibitors and statins [56–70].

From a drug safety perspective, emerging evidence emphasizes the need to assess the toxicity spectrum of CDK4/6 inhibitors in a more differentiated manner. Recent pharmacovigilance analyses indicate that adverse-event reporting is not uniform across palbociclib, ribociclib, and abemaciclib, reinforcing the importance of real-world data alongside clinical trial findings [70, 71]. The available data suggest that these agents differ not only in their toxicity profiles but also in their interaction liabilities, particularly in the context of CYP3A-mediated metabolism and transporter-related mechanisms. For example, ribociclib has been more consistently associated with QT prolongation and hepatotoxicity, abemaciclib with gastrointestinal toxicity, and palbociclib with predominantly hematologic adverse events, particularly neutropenia [71]. These differences may influence how interaction-related toxicities manifest in clinical practice, including in the setting of concomitant statin therapy. Recent clinical studies also suggest that concomitant medications may influence CDK4/6 inhibitor exposure and safety beyond CYP-mediated pathways, including through effects on drug absorption and patient-related factors [37, 72].

Post-marketing vigilance protocols are critical. The comprehensive use of adverse event reporting systems enables the identification of rare but severe toxicities, including those not evident in pivotal clinical trials. Importantly, pooled data from Phase III trials, such as MONALEESA-2, MONALEESA-3, and MONALEESA-7 for ribociclib, and PALOMA-1, PALOMA-2, and PALOMA-3 for palbociclib did not report significant incidences of rhabdomyolysis [73–77]. To date, this adverse reaction has emerged primarily in post-marketing settings involving complex polypharmacy.

Finally, multidisciplinary collaboration is critical to optimizing care in oncology patients receiving CYP3A4 inhibitors, such as the CDK4/6 inhibitors. Training programs should focus on the mechanisms of pharmacological interactions, practical strategies for preventing and managing adverse effects, and the importance of patient-centered care in complex clinical scenarios. Physicians and clinical pharmacists must align their knowledge to assess potential interactions and adjust therapies accordingly [78]. Clinical decision-support systems integrated into electronic health records could alert prescribers to high-risk combinations, further enhancing safety [79, 80]. Larger prospective clinical trials could help establish evidence-based protocols for managing these interactions.

From a methodological perspective, the quality appraisal checklist indicated an overall acceptable level of reporting across the included case reports. Most cases met key domains related to patient characterization, clinical course description, diagnostic assessment, and outcome reporting, supporting the internal consistency of the available evidence. Minor limitations were observed in selected reports, mainly involving incomplete descriptions of therapeutic interventions, post-intervention clinical status, or explicit clinical take-home messages [15, 16, 20]. These limitations are inherent to retrospective case-based evidence and do not undermine the coherence of the observed clinical patterns, but they should be considered when interpreting causality and generalizability.

This study has some limitations. Publication bias inherent to case-report–based evidence should be acknowledged, as more severe presentations tend to be preferentially reported, whereas milder cases may remain unrecognized or unpublished. Subclinical muscle toxicity is unlikely to be documented in case reports and therefore cannot be systematically captured, irrespective of search strategies. Given the small number of reports included in this review, the interpretations are limited. In addition, pharmacovigilance databases such as the FDA Adverse Event Reporting System (FAERS) or VigiBase may help identify further reports beyond the published case literature. These data, however, require cautious interpretation because duplicate reports, incomplete case information, and limited case-level causality assessment may affect their reliability. Although this review supports a causal link between CDK4/6 inhibitor-statin interactions and rhabdomyolysis, outcomes may have been influenced by concomitant substances such as fenugreek, comorbidities, and unrecognized drug-gene interactions. While one case explored pharmacogenetic predispositions [19], this was not uniformly investigated among the other included cases, leaving potential genetic contributions underexplored [15–18, 20, 21]. These caveats highlight the need for further research, including larger clinical studies and pharmacogenomic investigations. Furthermore, the analyzed cases may not reflect global clinical practices due to regional differences in prescribing patterns and patient management strategies.

## Conclusion

This systematic review identified seven reported cases of rhabdomyolysis associated with CDK4/6 inhibitor–statin interactions in patients with breast cancer, including one fatal case and one case with partial recovery. Although very rare, this adverse event can be severe; therefore, clinical vigilance is warranted, with close monitoring and early intervention if signs of muscle injury emerge. Further research and interdisciplinary collaboration are needed to optimize preventive and management strategies.

## Supplementary Information

Below is the link to the electronic supplementary material.

Supplementary Material 1

## Data Availability

No datasets were generated or analysed during the current study.

## Abbreviations

AKI: Acute kidney injury
CDK: Cyclin-dependent kinase
CDK4/6: Cyclin-dependent kinase 4 and 6
CK: Creatine kinase
CYP3A4: Cytochrome P450 3A4
ER: Estrogen receptor
HER2: Human epidermal growth factor receptor 2
HMG-CoA: Hydroxymethylglutaryl-CoA
IVIG: Intravenous immunoglobulin
OATP: Organic anion-transporting polypeptide
OATP1B1: Organic anion-transporting polypeptide 1B1
OATP1B3: Organic anion-transporting polypeptide 1B3
PCSK9: Proprotein convertase subtilisin/kexin type 9
PlEx: Plasma exchange
PRISMA: Preferred reporting items for systematic reviews and meta-analysis
SNP: Single nucleotide polymorphism
SLCO1B1: Solute carrier organic anion transporter family member 1B1
U/L: Units/liter
WHO-UMC: World health organization–uppsala monitoring centre
