# Supplementary material for: CDK4/6 inhibitor–statin interaction and rhabdomyolysis in breast cancer treatment: a case-based systematic review
Source: Cancer Chemother Pharmacol. 2026 Jul 1;96(1):67. doi: 10.1007/s00280-026-04913-w (PMC13319690; doi:10.1007/s00280-026-04913-w)
Supplement: Supplementary file 1 — Supplementary Material 1 [file 280_2026_4913_MOESM1_ESM.docx]

**Supplementary material**

| **Supplemental Table 1.** Inclusion and Exclusion Criteria Summary | |
| --- | --- |
| **Criteria** | **Details** |
| **Inclusion criteria** |  |
| Patient population | Breast cancer patients on CDK4/6 inhibitors and statins |
| Diagnosis of Rhabdomyolysis | CK > 1,000 U/L, clinical signs (e.g., weakness, dark urine), and/or AKI attributed to rhabdomyolysis |
| Data reporting | Sufficient data on demographics, drug regimens, clinical presentation, therapeutic interventions, and outcomes |
| Language | No restrictions; translation used as needed |
| Temporal scope | No publication date limits |
|  |  |
| **Exclusion criteria** |  |
| Incomplete data | Insufficient detail to link rhabdomyolysis to the CDK4/6 inhibitor–statin interaction |
| Duplicates | Only the most comprehensive report of duplicate cases included |
| Study type | Secondary analyses (e.g., reviews, editorials) excluded |
| CDK 4/6**:** Cyclin-dependent kinase 4/6; CK: Creatine Kinase; AKI: Acute Kidney Injury. | |

| **Supplemental Table 2.** Complete search strategy. | |
| --- | --- |
| PubMed, Embase, Scopus, and Web of Science | (Rhabdomyolysis OR Myopathy OR "Muscle damage") AND ("CDK4/6 Inhibitor" OR Palbociclib OR Ribociclib OR Abemaciclib) AND (Statins OR Simvastatin OR Atorvastatin OR Rosuvastatin OR Pravastatin OR Fluvastatin OR "HMG-CoA Reductase Inhibitors") |
